# Human Basigin (CD147) Does Not Directly Interact with SARS-CoV-2 Spike Glycoprotein

**DOI:** 10.1128/mSphere.00647-21

**Published:** 2021-08-11

**Authors:** Robert J. Ragotte, David Pulido, Francesca R. Donnellan, Michelle L. Hill, Giacomo Gorini, Hannah Davies, Juliane Brun, Kirsty McHugh, Lloyd D. W. King, Katherine Skinner, Kazutoyo Miura, Carole A. Long, Nicole Zitzmann, Simon J. Draper

**Affiliations:** a Jenner Institute, University of Oxford, Oxford, United Kingdom; b Department of Biochemistry, University of Oxford, Oxford, United Kingdom; c Oxford Glycobiology Institute, Department of Biochemistry, University of Oxford, Oxford, United Kingdom; d Laboratory of Malaria and Vector Research, National Institute of Allergy and Infectious Diseases, National Institutes of Health, Bethesda, Maryland, USA; University of Maryland School of Medicine

**Keywords:** CD147, COVID-19, SARS-CoV-2, basigin, coronavirus, virus entry, virus-host interactions

## Abstract

Basigin, or CD147, has been reported as a coreceptor used by severe acute respiratory syndrome coronavirus 2 (SARS-CoV-2) to invade host cells. Basigin also has a well-established role in Plasmodium falciparum malaria infection of human erythrocytes, where it is bound by one of the parasite’s invasion ligands, reticulocyte binding protein homolog 5 (RH5). Here, we sought to validate the claim that the receptor binding domain (RBD) of SARS-CoV-2 spike glycoprotein can form a complex with basigin, using RH5-basigin as a positive control. Using recombinantly expressed proteins, size exclusion chromatography and surface plasmon resonance, we show that neither RBD nor full-length spike glycoprotein bind to recombinant human basigin (expressed in either Escherichia coli or mammalian cells). Further, polyclonal anti-basigin IgG did not block SARS-CoV-2 infection of Vero E6 cells. Given the immense interest in SARS-CoV-2 therapeutic targets to improve treatment options for those who become seriously ill with coronavirus disease 2019 (COVID-19), we would caution the inclusion of basigin in this list on the basis of its reported direct interaction with SARS-CoV-2 spike glycoprotein.

**IMPORTANCE** Reducing the mortality and morbidity associated with COVID-19 remains a global health priority. Vaccines have proven highly effective at preventing infection and hospitalization, but efforts must continue to improve treatment options for those who still become seriously ill. Critical to these efforts is the identification of host factors that are essential to viral entry and replication. Basigin, or CD147, was previously identified as a possible therapeutic target based on the observation that it may act as a coreceptor for SARS-CoV-2, binding to the receptor binding domain of the spike protein. Here, we show that there is no direct interaction between the RBD and basigin, casting doubt on its role as a coreceptor and plausibility as a therapeutic target.

## INTRODUCTION

Since the emergence of severe acute respiratory syndrome coronavirus 2 (SARS-CoV-2) as the cause of the ongoing coronavirus disease 2019 (COVID-19) pandemic, there has been a rush to identify therapeutic targets that could reduce the immense human and economic toll of COVID-19. While vaccines are highly effective at preventing infection and hospitalization, for individuals who progress to severe disease there are limited, though improving, treatment options (1). Receptors required for viral entry are a natural consideration for druggable targets, as receptor blockade could both prevent infection if a drug is delivered prophylactically and treat infection by impeding viral replication to reduce viral load. Moreover, there may be existing monoclonal antibodies (MAbs) approved for clinical use that target these receptors.

After the release of the genome sequence of SARS-CoV-2, the primary entry receptor was rapidly identified as angiotensin-converting enzyme 2 (ACE2) (2–6). This is the same entry receptor used by some other coronaviruses, most notably SARS-CoV, a highly similar coronavirus that emerged in 2002 (2, 7). Since this initial identification of ACE2, there has been significant discussion in the literature, both peer-reviewed and preprint, about other coreceptors or cofactors required for entry (6, 8–10). Transmembrane protease, serine 2 (TMPRSS2), is one such cofactor that has been identified and subsequently validated by multiple groups, which cleaves the spike protein to facilitate entry (6, 11, 12).

CD147, or basigin, was first proffered as a spike glycoprotein coreceptor in preprint literature in March 2020, which has since been published in a peer-reviewed journal (10). The authors showed that spike binding to basigin has important functional implications for viral entry, making basigin blockade an attractive therapeutic target (10). Since this initial finding, basigin has been included in discussions of SARS-CoV-2 coreceptors (8, 13–21).

Basigin is ubiquitously expressed in human tissues and forms a complex with monocarboxylate transporters (MCTs) and the glucose transporter GLUT1, among others (22). In the context of infectious disease, basigin has also been well characterized as an essential receptor for Plasmodium falciparum invasion into human erythrocytes, during which it is bound by the malaria parasite’s reticulocyte-binding protein homolog 5 (RH5) (23, 24). Based on the initial observation that appeared to show that basigin binds to the SARS-CoV-2 spike glycoprotein receptor binding domain (RBD) (10), clinical trials were initiated investigating an anti-basigin MAb as a therapeutic for COVID-19 (30) (ClinicalTrials.gov identifier NCT04275245).

Having worked extensively with basigin in the context of its RH5 interaction, we aimed to validate the finding that basigin directly interacts with the receptor binding domain of the SARS-CoV-2 spike protein. Here, we show that we could not replicate this finding. Although we saw clear binding of recombinant SARS-CoV-2 full-length spike trimer (FL-S) and RBD to ACE2 and the anti-RBD MAb CR3022 (26), we did not see any binding to glycosylated or nonglycosylated basigin by size exclusion chromatography (SEC) or surface plasmon resonance (SPR). Meanwhile, recombinant RH5 showed clear binding to both glycosylated and nonglycosylated basigin by the same methods. Finally, we show that anti-basigin polyclonal rabbit IgG can potently inhibit *in vitro* growth of malaria parasites but has no impact on SARS-CoV-2 infection of Vero E6 cells. In sum, this evidence does not support a role of basigin in SARS-CoV-2 infection.

## RESULTS

### Spike and RBD bind human ACE2 via SEC.

**FIG 1:**
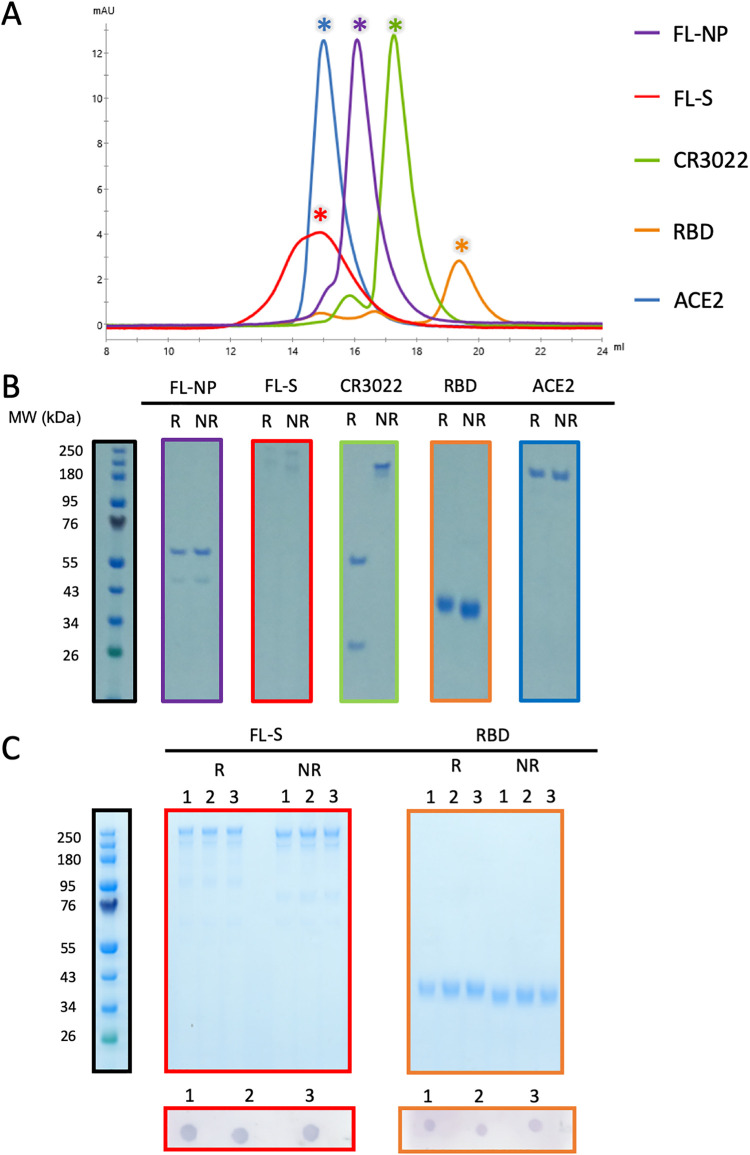
(A) Size exclusion chromatograms postpurification of FL-NP, FL-S, CR3022, RBD, and ACE2. All proteins were run individually with chromatograms overlaid. Asterisks indicate the fractions run on SDS-PAGE. (B) Nonreducing (NR) and reducing (R) Coomassie blue-stained SDS-PAGE protein gels of 1 μg of protein from the asterisk-indicated fractions. (C) Freeze-thaw stability of FL-S and RBD. Reducing and nonreducing SDS-PAGE protein gel of 1 μg FL-S (red outline) and RBD (orange outline) after 1, 2, and 3 freeze-thaw cycles. Below each gel a dot blot is shown, using the CR3022 human MAb on 1 μg FL-S and RBD after 1, 2, and 3 freeze-thaw cycles.

Initially we produced a panel of recombinant protein reagents. Recombinant human ACE2, SARS-CoV-2 full-length spike trimer (FL-S), full-length nucleoprotein (FL-NP), spike RBD, and anti-RBD antibody CR3022 were all expressed by transient transfection in mammalian Expi293 cells (Fig. 1A and B). Glycosylated and nonglycosylated basigin were expressed in Expi293 cells and Escherichia coli, respectively, and glycosylation states were confirmed by PNGase F digest (Fig. S1). Correct folding of RBD and FL-S was confirmed via dot blotting using CR3022, a known SARS-CoV-2 RBD- and FL-S-binding MAb (27), as the primary antibody (Fig. 1C). These data showed that all proteins were expressed as expected and demonstrated high levels of purity. The FL-S and RBD also showed stability upon freeze-thawing and retained binding of the conformation-sensitive MAb CR3022 after three freeze-thaw cycles (Fig. 1C).

10.1128/mSphere.00647-21.1 FIG S1 PNGase F digest of E. coli-expressed (nonglycosylated) and Expi293-expressed (glycosylated) basigin. The lower molecular weight of glycosylated basigin after PNGase F treatment is consistent with the loss of glycans. The higher molecular weight of glycosylated basigin treated with PNGase F compared to nonglycosylated basigin can be attributed to the presence of the rat CD4 domain 3 and 4 (CD4d3+4) solubility tag (33 kDa). Download FIG S1, TIF file, 1.7 MB. Copyright © 2021 Ragotte et al. 2021 Ragotte et al. https://creativecommons.org/licenses/by/4.0/ This content is distributed under the terms of the Creative Commons Attribution 4.0 International license.

**FIG 2:**
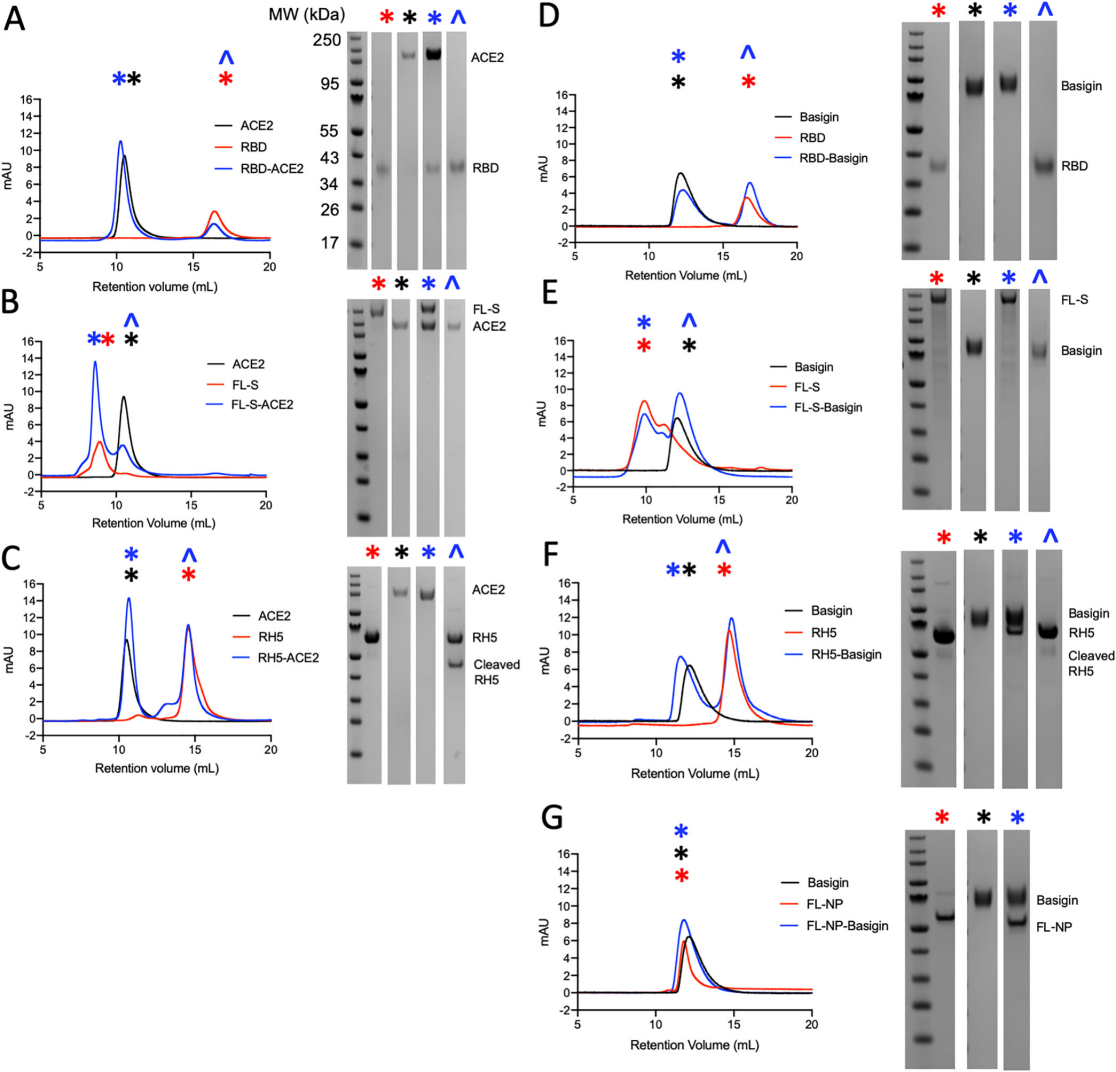
Size exclusion chromatograms (left) and accompanying SDS-PAGE gels (right) assessing complex formation between RH5/RBD/FL-S/RBD and ACE2/basigin. Symbols on chromatograms indicate which gels correspond to the peaks. Full-length RH5 (∼60 kDa) undergoes cleavage at room temperature to yield an ∼43-kDa band. (A) RBD-ACE2; (B) FL-S-ACE2; (C) RH5-ACE2; (D) RBD-basigin; (E) FL-S–basigin; (F) RH5-basigin; (G) FL-NP–basigin.

We next confirmed SARS-CoV-2 RBD and FL-S binding to human ACE2 using SEC (Fig. 2A). When RBD and ACE2 were incubated together, the complex eluted at an earlier retention volume than ACE2 alone, indicative of the formation of a higher-molecular-weight complex. Complex formation was then confirmed using SDS-PAGE, whereby both RBD and ACE2 eluted within the same peak at approximately 10 ml, whereas RBD alone normally elutes at approximately 16 ml (Fig. 2A).

This was next confirmed in the same manner with FL-S trimer, which also eluted as a complex with ACE2 when they were incubated together, as shown by SDS-PAGE (Fig. 2B). Although there was only a small change in retention volume between FL-S alone and the FL-S-ACE2 complex, this can be attributed to the use of an S200 column, whose resolution limits are less than the expected size of the FL-S-ACE2 complex (approximately 680 kDa). Nonetheless, it is clear that ACE2 eluted with FL-S at an approximately 8-ml retention volume, while ACE2 alone eluted at approximately 11 ml (Fig. 2B). We next demonstrated there was no interaction between the RH5 malaria antigen and ACE2 (as expected), given that both proteins eluted at the same retention volume whether alone or mixed together (Fig. 2C). These results confirm that our recombinant FL-S, RBD, and ACE2 demonstrate the established interactions.

### SARS-CoV-2 spike and RBD do not bind human basigin via SEC.

Having confirmed the interaction between FL-S/RBD and ACE2, we proceeded to assess FL-S and RBD binding to glycosylated human basigin using the same method. RH5, which acted as the positive control, showed clear binding to basigin, forming a stable complex in solution, as confirmed by SEC and SDS-PAGE (Fig. 2F). The binding affinity between RH5 and basigin is weaker than the reported values for RBD and basigin (approximately 1 μM for RH5 [23, 24] compared to 185 nM for RBD [10]), indicating that this assay should be sufficiently sensitive to detect the RBD-basigin interaction.

SARS-CoV-2 FL-NP was used as a negative control and did not form a complex with basigin. Coincidentally, both FL-NP and basigin elute at the same retention volume, but the absence of any shift to a higher-molecular-weight complex when they are incubated together is consistent with no complex formation (Fig. 2G). Next, we observed that there was no detectable binding between either RBD or FL-S and glycosylated basigin, with both RBD and FL-S eluting separately from basigin (Fig. 2D and E). Thus, it did not appear that any complex could be formed in solution between these proteins.

Finally, in order to confirm whether glycosylation may affect binding, we performed the experiment again using the basigin ectodomain expressed in E. coli (Fig. S1), as described by Wright et al. (23). Again, there was clear binding to RH5 but no discernible binding to either the FL-S or RBD protein (Fig. S2).

10.1128/mSphere.00647-21.2 FIG S2 Size exclusion chromatograms (left) and accompanying SDS-PAGE gels (right) of nonglycosylated basigin binding to RH5/RBD/FL-S. (A) RH5-basigin; (B) RBD-basigin; (C) FL-S–basigin. Download FIG S2, TIF file, 2.7 MB. Copyright © 2021 Ragotte et al. 2021 Ragotte et al. https://creativecommons.org/licenses/by/4.0/ This content is distributed under the terms of the Creative Commons Attribution 4.0 International license.

### SARS-CoV-2 spike and RBD do not bind to human basigin via SPR.

**FIG 3:**
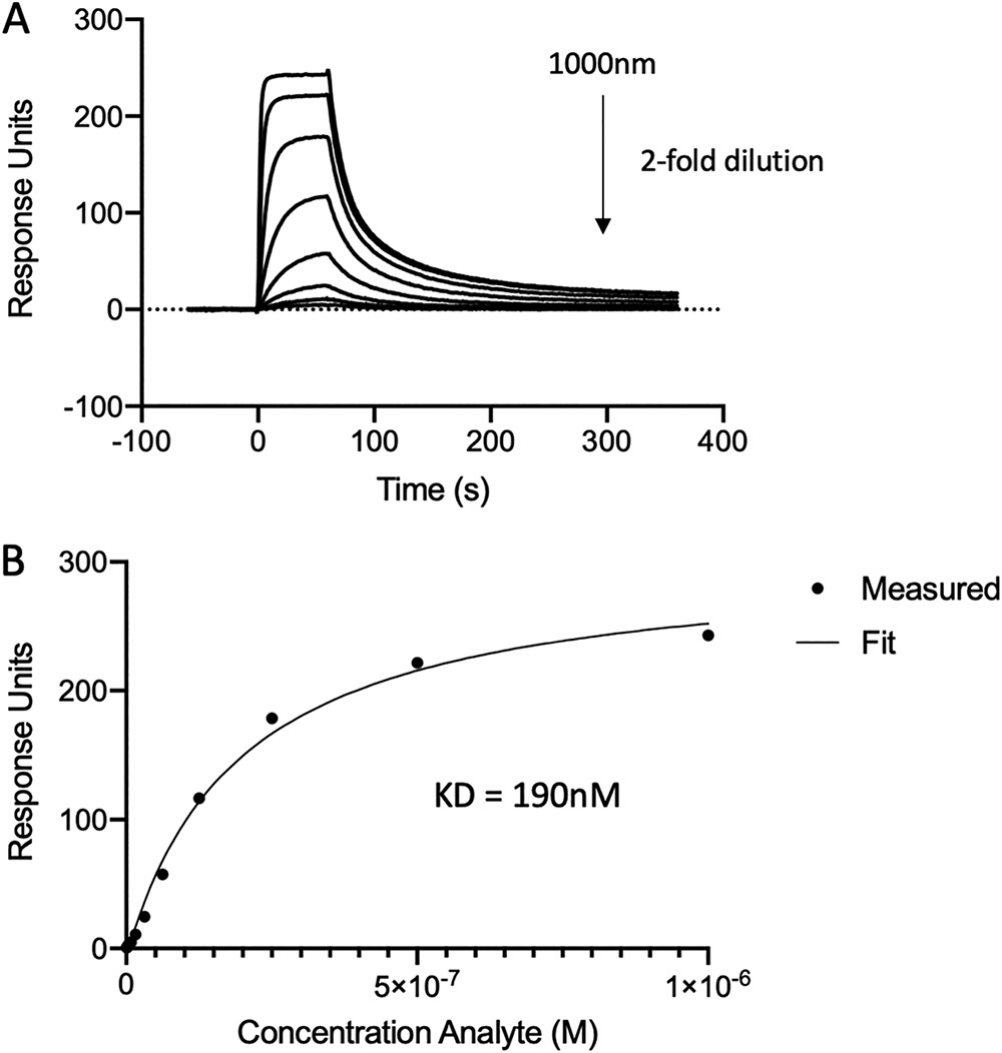
Steady-state affinity of CR3022 MAb binding to RBD as assessed using SPR. (A) Sensorgram of an 8-step dilution curve beginning at 1 μM. (B) Calculation of steady-state affinity.

Although it was clear that the reported FL-S/RBD-basigin complex was not stable enough to detect via SEC, we next sought to confirm the previously reported SPR data showing the RBD-basigin interaction (10). To begin, we confirmed that the RBD protein interacted with CR3022 with the expected affinity, via an 8-step dilution curve beginning at 1 μM. The steady-state affinity was determined to be 190 nM, consistent with published data on this interaction (26) (Fig. 3A and B).

**FIG 4:**
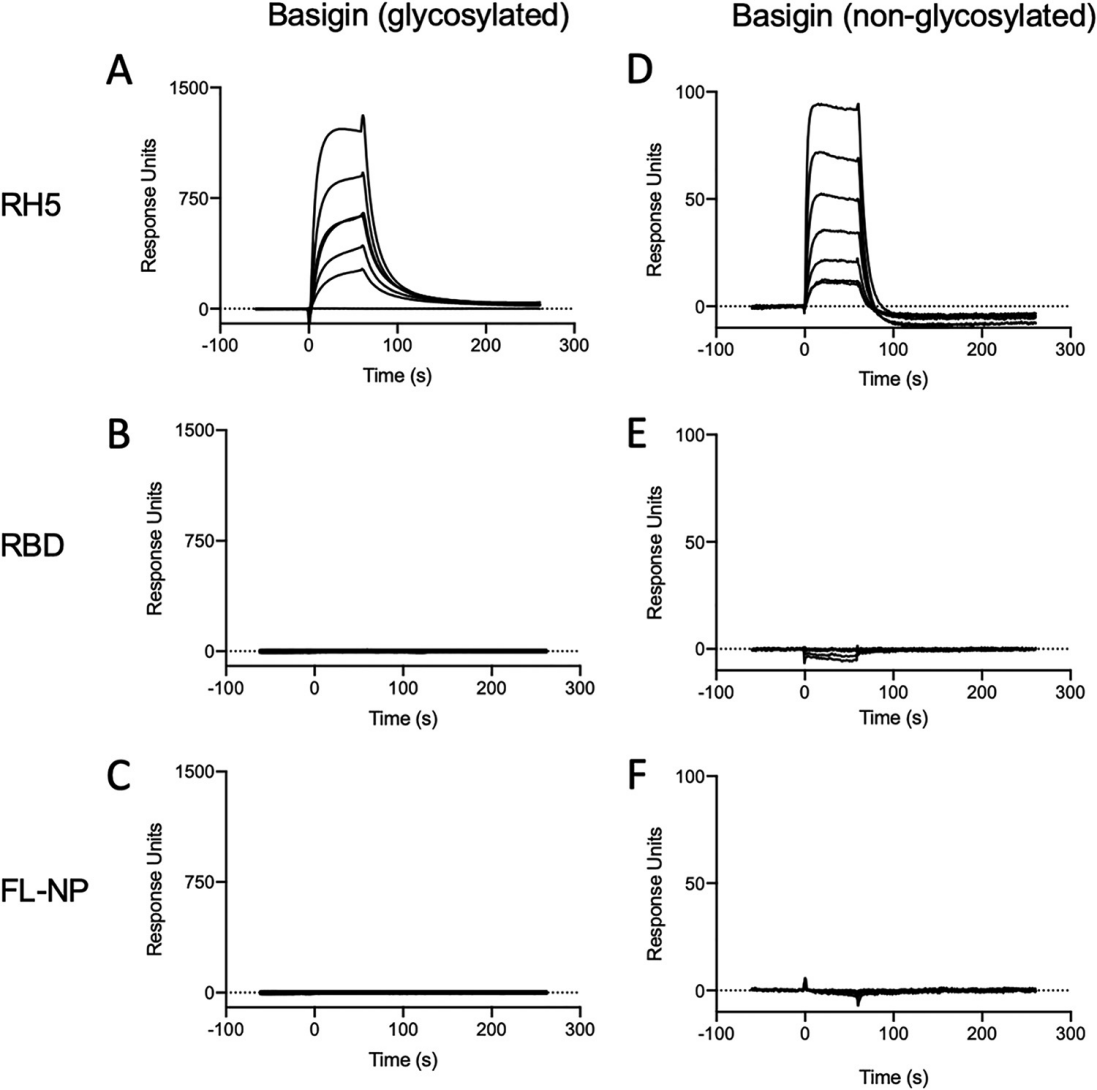
SPR analysis of protein binding interactions. Sensorgrams show binding of each protein to either glycosylated or nonglycosylated basigin (coupled to the chip). Protein binding was assessed along 5-step 2-fold dilution curves starting at 2 μM. (A) Glycosylated basigin binding to RH5; (B) glycosylated basigin binding to RBD; (C) glycosylated basigin binding to FL-NP; (D) nonglycosylated basigin binding to RH5; (E) nonglycosylated basigin binding to RBD; (F) nonglycosylated basigin binding to FL-NP.

Next, basigin, either glycosylated (Fig. 4A to C) or nonglycosylated (Fig. 4D to F), was immobilized through amine conjugation on a CM5 chip. RH5, RBD, or FL-NP was then flowed over the chip to determine binding and affinity. RH5 clearly bound to both forms of basigin with steady-state affinities of approximately 925 ± 16 nM for bacterially expressed basigin and 665 ± 39 nM for mammalian cell-expressed basigin, in line with previous reports (23, 24) (Fig. 4A and D). However, RBD did not show any discernible binding to either glycosylated (Fig. 4B) or nonglycosylated basigin (Fig. 4E). FL-NP also did not bind to either form of basigin, as expected (Fig. 4C and F).

### Anti-basigin polyclonal IgG does not block SARS-CoV-2 infection of Vero E6 cells.

**FIG 5:**
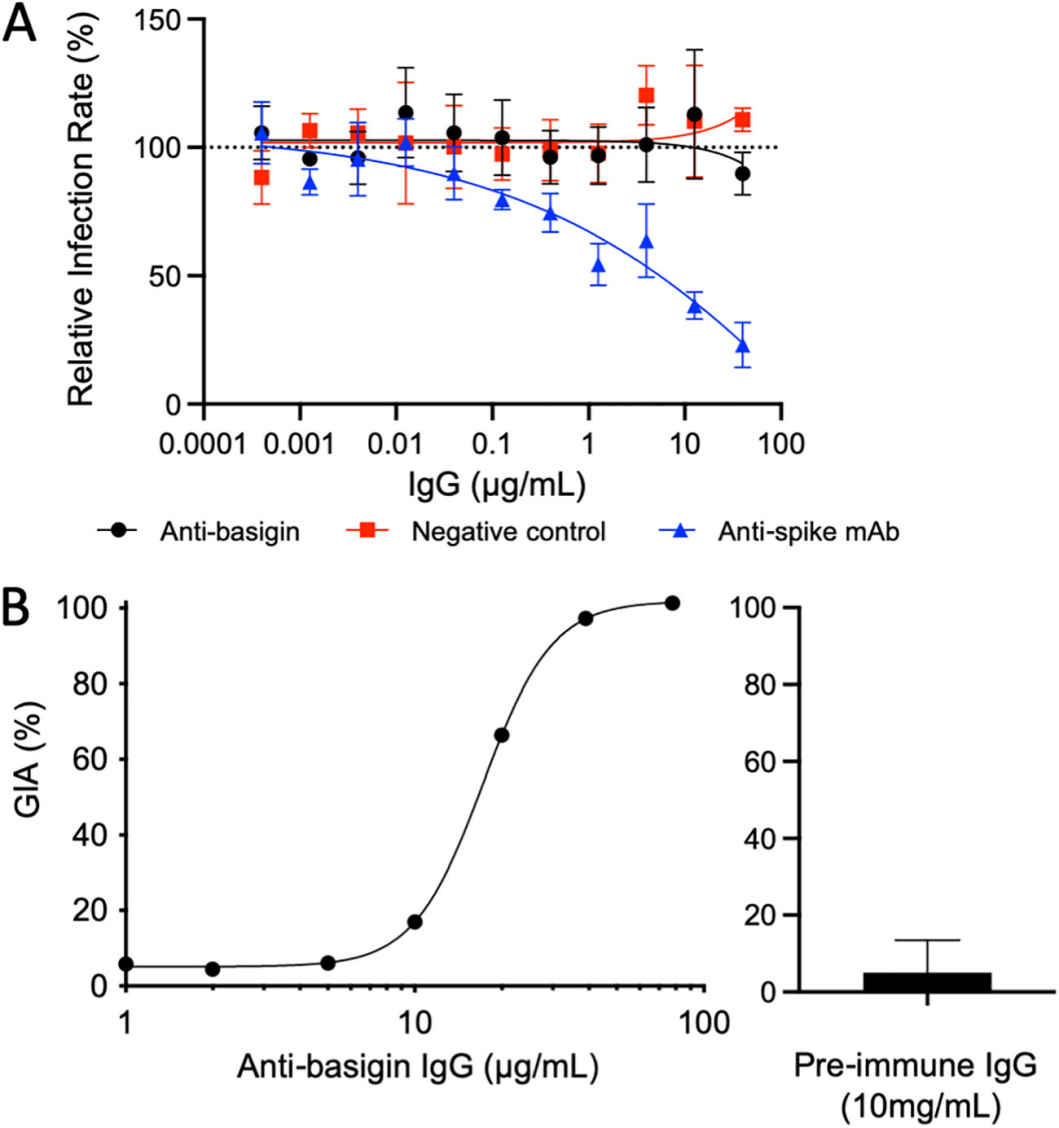
(A) Virus neutralization assay assessing clinical SARS-CoV-2 isolate SARS-CoV-2/human/Eng/2-20 infection of Vero E6 cells in the presence of purified IgG from human basigin-immunized rabbits (black). All values are relative to a virus-only control (dotted line at *y* = 100). Each point indicates the mean, and error bars show standard deviations of triplicate measurement. The negative control was purified IgG from rabbits vaccinated with S antigen (red). The positive control was the published inhibitory anti-spike MAb 1B10 (blue) (B) (Left) P. falciparum assay of growth inhibition activity (GIA) showing the functionality of the purified IgG from basigin-immunized rabbits, where each point is the mean of a triplicate. (Right) Purified IgG prior to immunization with basigin, tested for GIA at 10 mg/ml (*n* = 2). The bar indicates the mean, and the error bar shows the standard deviation.

In order to rule out the possibility that basigin is indirectly involved in the SARS-CoV-2 invasion process, we measured neutralization of SARS-CoV-2 infection of Vero E6 cells by anti-human basigin polyclonal IgG. We purified polyclonal IgG from rabbits immunized with human basigin and conducted a viral neutralization assay (Fig. 5A). Polyclonal anti-basigin IgG showed no effect on virus neutralization, closely matching the negative-control IgG. As a positive control, we also tested a previously published neutralizing anti-spike MAb, 1B10 (28), which prevented infection in a dose-dependent manner (Fig. 5A).

To ensure that the anti-basigin serum was functional, the same reagent was used to measure inhibition of P. falciparum invasion. This clearly demonstrated the functionality of the anti-basigin rabbit IgG, with a 50% effective concentration (EC_50_) of 17 μg/ml, while the preimmune IgG did not show any neutralizing activity even at 10 mg/ml (Fig. 5B). At 40 μg/ml, the same top concentration used in the viral neutralization assays, 97.3% growth inhibition activity (GIA) was observed (Fig. 5B). These data confirm previously reported results showing that knockdown of basigin did not block viral entry in a CaLu-3 cell viral invasion assay (29), both in contradiction to the original observation that anti-basigin MAbs could neutralize SARS-CoV-2 *in vitro* (10).

## DISCUSSION

Here, we show that neither SARS-CoV-2 RBD nor full-length spike trimer binds to recombinant human basigin. Further, anti-basigin polyclonal IgG did not inhibit viral entry. This is in contrast to a previous report which identified basigin as a coreceptor for SARS-CoV-2, showed binding of RBD to spike via SPR assays and ELISA, and showed that meplazumab, an anti-basigin MAb, could neutralize SARS-CoV-2 (10). The use of an anti-basigin MAb in clinical trials has begun on the basis of the original observation that basigin may be required for host cell entry (30). We believe that it is necessary to proceed with caution when interpreting the trial data, as further investigation is warranted to determine what role, if any, basigin has in the SARS-CoV-2 invasion process.

Our findings here are also supported by another independent investigation (29). Shilts et al. also showed evidence that there is no direct interaction between CD147 and full-length spike or its S1 domain using a different set of methods than used here (avidity-based extracellular interaction screening and tetramer-staining of HEK293 cells expressing basigin) (29). Their studies complement the work described here, as they also evaluated this interaction using two different isoforms of basigin and in a cellular invasion assay (29), whereas here we evaluated only the far more abundant basigin-2 isoform (31).

Initial evaluation of meplazumab, an anti-basigin antibody, for treatment of SARS-CoV-2 pneumonia suggested that there could be a benefit (30). These results are based on small groups (*n* = 17 in the treatment group) (30); as such, it will be necessary to conduct larger clinical studies to confirm this finding. If indeed these findings hold, the reported effect could be due to a nonspecific anti-inflammatory effect of basigin blockade, as there have been some reports of a proinflammatory role of basigin in immune signaling (32–36). Any benefit of meplazumab is thus unlikely to be due to the direct inhibition of viral entry, due to the fact that SARS-CoV-2 spike protein does not appear to interact with basigin in our study or that of Shilts et al. (29).

Nevertheless, the data surrounding the safety and tolerability of anti-basigin may have implications beyond SARS-CoV-2, as these trials could inform the use of anti-basigin as a malaria prophylactic regardless of its effectiveness in reducing mortality and morbidity due to COVID-19. To date, this has not been pursued in the malaria field beyond *in vitro* assays (37) or humanized mouse models (38), despite demonstration of remarkable potency of anti-basigin MAbs, further supported here by our GIA assay data using the same polyclonal anti-basigin IgG against P. falciparum. This is largely due to safety concerns regarding prophylactics that would target a human host protein, as opposed to the parasite, in a vulnerable or infant target population. Should this therapy prove to be safe and well tolerated, it could be further explored in malaria, where basigin has a well-established role in pathogen invasion.

### Conclusion.

Recombinant basigin (CD147) does not bind directly to the SARS-CoV-2 RBD. The data presented here do not support the role of basigin as a possible SARS-CoV-2 coreceptor.

## MATERIALS AND METHODS

### Recombinant protein expression and purification.

A construct for soluble trimeric spike (FL-S) glycoprotein of SARS-CoV-2 (NCBI reference sequence YP_009724390.1), encoding residues M1 to P1213 with two sets of mutations that stabilize the protein in a prefusion conformation (removal of a furin cleavage site and the introduction of two proline residues, K986P and V987P), was expressed as described previously (39). This construct includes the endogenous viral signal peptide at the N terminus (residues 1 to 14), while a C-terminal T4-foldon domain is incorporated to promote association of monomers into trimers to reflect the native transmembrane viral protein. The RBD construct utilized the native SARS-CoV-2 spike signal peptide (residues 1 to 14) fused directly to residues R319 to F541 of the spike glycoprotein, which encompasses the binding site for the human receptor ACE2 (39). Both constructs include a C-terminal hexahistidine (His6) tag for nickel-based affinity purification. FL-S and RBD were transiently expressed in Expi293 cells (Thermo Fisher Scientific) and protein purified from culture supernatants by immobilized metal affinity followed by gel filtration in Tris-buffered saline (TBS) (pH 7.4) buffer.

Full-length SARS-CoV-2 nucleoprotein (FL-NP; NCBI reference sequence YP_009724397.2; residues M1 to A419) was transiently expressed in Expi293 cells (Thermo Fisher Scientific) intracellularly. The FL-NP construct included a C-tag peptide (EPEA) at the C terminus (40) for affinity chromatography purification using a C-tag affinity resin (Thermo Fisher Scientific), eluting with 20 mM Tris-HCl, 2 M MgCl_2_ (pH 7.4). Affinity purification was followed by size exclusion chromatography in TBS (pH 7.4) buffer. Human ACE2 ectodomain (NCBI reference sequence NP_001358344.1; residues Q18 to S740) was expressed in Expi293 cells (Thermo Fisher Scientific) with a preceding murine IgG1 signal peptide, mono-Fc domain, and tobacco etch virus (TEV) cleavage site at the N terminus and a GTGGS flexible linker and C-tag peptide at the C terminus. ACE2-containing supernatant was purified by C-tag affinity followed by size exclusion chromatography in TBS (pH 7.4) buffer. Human IgG1 CR3022 antibody (27) (GenBank accession no. ABA54613.1 and ABA54614.1) was expressed from heavy- and light-chain AbVec expression vectors in Expi293 cells (Thermo Fisher Scientific). CR3022 supernatant was purified using HiTrap protein G HP (Cytiva) followed by size exclusion chromatography in TBS (pH 7.4) buffer. Wild-type/native human basigin (BSG-2; residues M1 to L206) was expressed in Expi293 cells (Thermo Fisher Scientific) with a tag containing C-terminal rat CD4 domains 3 and 4 (CD4d3+4) (to aid expression and solubility), followed by a His6 tag for purification, as described previously (24, 41). Nonglycosylated basigin (also BSG-2) was expressed in Escherichia coli with an N-terminal His6 tag followed by a TEV cleavage site, as described previously (23). The recombinant RH5 sequence is based on the P. falciparum 3D7 clone reference sequence and encodes amino acids E26 to Q526. The construct includes a C-terminal C tag and four mutations to delete N-linked glycosylation sequons (T40A, T216A, T286A, and T299A). This construct was expressed as a secreted protein by a stable *Drosophila* S2 cell line (42) and affinity purified using C-tag affinity resin (Thermo Fisher Scientific) followed by a size exclusion chromatography polishing step in 20 mM Tris, 150 mM NaCl (pH 7.4).

### SEC binding assay.

One hundred micrograms of recombinant receptor (either mammalian- or E. coli*-*expressed basigin or ACE2) was mixed in a 1:1 molar ratio with RH5, FL-S, or RBD and then incubated for 1 h at room temperature (RT). After incubation, samples were loaded onto a S200 10/300 column via direct injection using an Äkta Pure (GE Healthcare) and run at 0.8 ml/min at RT. Eluted fractions were collected and run on SDS-PAGE under reducing or nonreducing conditions before staining with Coomassie blue.

### Protein blots.

Samples were diluted 1:4 in Laemmli buffer, with or without dithiothreitol (DTT), and then heated at 95°C for 5 min before loading onto a precast 4 to 12% bis-Tris polyacrylamide gel (Thermo Fisher Scientific). Samples were run at 200 V for 45 min before staining with Coomassie blue.

One microgram of FL-S or RBD was pipetted onto a 0.2-μm nitrocellulose membrane and allowed to air dry. Immunoblotting was performed using the Invitrogen iBind Western system according to the manufacturer’s instructions. CR3022 was used as a primary antibody diluted to 2 μg/ml. Alkaline phosphatase-conjugated goat anti-human IgG, Fc-specific (Sigma), diluted to 1:2,000 was used for detection with Sigmafast BCIP/NBT alkaline phosphatase substrate at 1 mg/ml (Sigma-Aldrich).

### Surface plasmon resonance.

Basigin, expressed in either mammalian cells or E. coli, was immobilized on a CM5 chip through amine conjugation using NHS/EDC coupling using a Biacore X100 system (GE Healthcare). Samples were run at 30 μl/min with an injection time of 60 s and a dissociation of 200 s. Then, 5-step 2-fold dilution curves of either RH5, RBD, or FL-NP were run starting at 2 μM, with regeneration of the chip via injection of 10 mM glycine (pH 2) for 30 s. Between runs, a single injection of RH5 at 2 μM was carried out to confirm that there was no loss in binding activity. For CR3022 binding affinity, approximately 400 response units (RU) of antibody was captured on a protein A chip. Steady-state affinity was determined through an 8-step dilution curve beginning at 1 μM, with 10 mM glycine (pH 2) used to regenerate the chip between curves. All curves included one duplicate concentration and were evaluated using the Biacore X100 evaluation software.

### PNGase F treatment.

PNGase F treatment was conducted per the manufacturer’s protocol (New England Biolabs). Briefly, 10 μl of basigin at 0.5 μg/μl expressed in either E. coli or Expi293 cells underwent denaturation at 95°C for 10 min in glycoprotein denaturing buffer (New England Biolabs) followed by immediate cooling on ice for 10 s. Then, the denatured protein was mixed with 2 μl of GlycoBuffer 2, 2 μl 10% NP-40, 6 μl of water, and 1 μl of PNGase F. After incubation for 1 h at 37°C, samples were analyzed by nonreducing SDS-PAGE and stained with Coomassie blue.

### Anti-basigin polyclonal serum generation.

Work using rabbits was reviewed by the University of Oxford Animal Welfare and Ethical Review Board and conducted by Cambridge Research Biochemicals, UK, in accordance with the terms of the UK Animals (Scientific Procedures) Act Project Licence. A New Zealand White rabbit (Cambridge Research Biochemicals, UK) was immunized with 100 μg recombinant human basigin or P. falciparum S antigen (negative control) in 50% (vol/vol) (200 μl total) AddaVax adjuvant (InvivoGen) on days 0, 28, and 56, prior to serum harvest on day 63. Total IgG was purified from serum using protein G columns (Thermo Fisher). Serum was diluted 1:1 in IgG binding buffer (Thermo Fisher) and loaded onto an equilibrated protein G column. The IgG-bound column was then washed with phosphate-buffered saline (PBS), and IgG was eluted with 0.1 M glycine (pH 2) into 1 M Tris HCl (pH 9) to reach a pH of 7.4.

### Viral neutralization assay.

The SARS-CoV-2 isolate SARS-CoV-2/human/Eng/2-20 was provided at passage 1 from Public Health England and was propagated in Vero E6 cells in Dulbecco’s modified Eagle medium (DMEM) with 2% heat-inactivated fetal bovine serum (FBS), 100 U/ml penicillin, and 0.1 mg/ml streptomycin (MilliporeSigma). Antibody neutralization potential was determined using a focus reduction neutralization test (FRNT). Half-log serial dilutions of antibody (purified rabbit IgG) were mixed with SARS-CoV-2 harvested 72 h postinfection (multiplicity of infection = 0.01) and incubated at RT for 30 min before adding to Vero E6 cells and incubating for 2 h at 37°C and 5% CO_2_. semisolid carboxymethyl cellulose (CMC) (2%; Merck) overlay medium (1:1 mix of 4% CMC in H_2_O and virus propagation medium) was then added to each well, and plates were incubated for 20 h at 37°C and 5% CO_2_. After incubation, cell monolayers were fixed with 4% paraformaldehyde and then stained with human anti-N MAb (EY2A) followed by peroxidase-conjugated goat anti-human IgG (A0170; Sigma) and visualized using TrueBlue peroxidase substrate (50-78-02; Insight Biotechnologies). Foci were counted using an AID Classic enzyme-linked immunosorbent spot (ELISpot) assay reader (Autoimmun Diagnostika GmbH).

### P. falciparum assay of growth inhibition activity.

Assays of growth inhibition activity (GIA) were done at the GIA Reference Centre, NIAID, NIH, as described previously (43). Briefly, one-cycle GIA was done at the indicated concentration of purified rabbit IgG using tightly synchronized cultures of late trophozoites/schizonts prepared using 5% sorbitol treatment. Invasion of red blood cells was quantified using a P. falciparum lactate dehydrogenase assay. Invasion was reported relative to medium alone. All samples were run in triplicate.

### Data availability.

Requests for materials should be addressed to the corresponding author.

## References

[1] Stasi C, Fallani S, Voller F, Silvestri C. 2020. Treatment for COVID-19: an overview. Eur J Pharmacol 889:173644. doi:10.1016/j.ejphar.2020.173644. 33053381 PMC7548059

[2] Letko M, Marzi A, Munster V. 2020. Functional assessment of cell entry and receptor usage for SARS-CoV-2 and other lineage B betacoronaviruses. Nat Microbiol 5:562–569. doi:10.1038/s41564-020-0688-y. 32094589 PMC7095430

[3] Shang J, Ye G, Shi K, Wan Y, Luo C, Aihara H, Geng Q, Auerbach A, Li F. 2020. Structural basis of receptor recognition by SARS-CoV-2. Nature 581:221–224. doi:10.1038/s41586-020-2179-y. 32225175 PMC7328981

[4] Yan R, Zhang Y, Li Y, Xia L, Guo Y, Zhou Q. 2020. Structural basis for the recognition of SARS-CoV-2 by full-length human ACE2. Science 367:1444–1448. doi:10.1126/science.abb2762. 32132184 PMC7164635

[5] Wang Q, Zhang Y, Wu L, Niu S, Song C, Zhang Z, Lu G, Qiao C, Hu Y, Yuen K-Y, Wang Q, Zhou H, Yan J, Qi J. 2020. Structural and functional basis of SARS-CoV-2 entry by using human ACE2. Cell 181:894–904.E9. doi:10.1016/j.cell.2020.03.045. 32275855 PMC7144619

[6] Hoffmann M, Kleine-Weber H, Schroeder S, Krüger N, Herrler T, Erichsen S, Schiergens TS, Herrler G, Wu N-H, Nitsche A, Müller MA, Drosten C, Pöhlmann S. 2020. SARS-CoV-2 cell entry depends on ACE2 and TMPRSS2 and is blocked by a clinically proven protease inhibitor. Cell 181:271–280.E8. doi:10.1016/j.cell.2020.02.052. 32142651 PMC7102627

[7] Li W, Moore MJ, Vasilieva N, Sui J, Wong SK, Berne MA, Somasundaran M, Sullivan JL, Luzuriaga K, Greenough TC, Choe H, Farzan M. 2003. Angiotensin-converting enzyme 2 is a functional receptor for the SARS coronavirus. Nature 426:450–454. doi:10.1038/nature02145. 14647384 PMC7095016

[8] Ng KW, Faulkner N, Cornish GH, Rosa A, Harvey R, Hussain S, Ulferts R, Earl C, Wrobel AG, Benton DJ, Roustan C, Bolland W, Thompson R, Agua-Doce A, Hobson P, Heaney J, Rickman H, Paraskevopoulou S, Houlihan CF, Thomson K, Sanchez E, Shin GY, Spyer MJ, Joshi D, O'Reilly N, Walker PA, Kjaer S, Riddell A, Moore C, Jebson BR, Wilkinson M, Marshall LR, Rosser EC, Radziszewska A, Peckham H, Ciurtin C, Wedderburn LR, Beale R, Swanton C, Gandhi S, Stockinger B, McCauley J, Gamblin SJ, McCoy LE, Cherepanov P, Nastouli E, Kassiotis G. 2020. Preexisting and de novo humoral immunity to SARS-CoV-2 in humans. Science 370:1339–1343. doi:10.1126/science.abe1107. 33159009 PMC7857411

[9] Herrera NG, Morano NC, Celikgil A, Georgiev GI, Malonis RJ, Lee JH, Tong K, Vergnolle O, Massimi AB, Yen LY, Noble AJ, Kopylov M, Bonanno JB, Garrett-Thomson SC, Hayes DB, Bortz RH, III, Wirchnianski AS, Florez C, Laudermilch E, Haslwanter D, Fels JM, Dieterle ME, Jangra RK, Barnhill J, Mengotto A, Kimmel D, Daily JP, Pirofski LA, Chandran K, Brenowitz M, Garforth SJ, Eng ET, Lai JR, Almo SC. 2021. Characterization of the SARS-CoV-2 S protein: biophysical, biochemical, structural, and antigenic analysis. ACS Omega 6:85–102. doi:10.1021/acsomega.0c03512. 33458462 PMC7771249

[10] Wang K, Chen W, Zhang Z, Deng Y, Lian J-Q, Du P, Wei D, Zhang Y, Sun X-X, Gong L, Yang X, He L, Zhang L, Yang Z, Geng J-J, Chen R, Zhang H, Wang B, Zhu Y-M, Nan G, Jiang J-L, Li L, Wu J, Lin P, Huang W, Xie L, Zheng Z-H, Zhang K, Miao J-L, Cui H-Y, Huang M, Zhang J, Fu L, Yang X-M, Zhao Z, Sun S, Gu H, Wang Z, Wang C-F, Lu Y, Liu Y-Y, Wang Q-Y, Bian H, Zhu P, Chen Z-N. 2020. CD147-spike protein is a novel route for SARS-CoV-2 infection to host cells. Signal Transduct Target Ther 5:283. doi:10.1038/s41392-020-00426-x. 33277466 PMC7714896

[11] Matsuyama S, Nao N, Shirato K, Kawase M, Saito S, Takayama I, Nagata N, Sekizuka T, Katoh H, Kato F, Sakata M, Tahara M, Kutsuna S, Ohmagari N, Kuroda M, Suzuki T, Kageyama T, Takeda M. 2020. Enhanced isolation of SARS-CoV-2 by TMPRSS2-expressing cells. Proc Natl Acad Sci USA 117:7001–7003. doi:10.1073/pnas.2002589117. 32165541 PMC7132130

[12] Zang R, Castro MFG, McCune BT, Zeng Q, Rothlauf PW, Sonnek NM, Liu Z, Brulois KF, Wang X, Greenberg HB, Diamond MS, Ciorba MA, Whelan SPJ, Ding S. 2020. TMPRSS2 and TMPRSS4 promote SARS-CoV-2 infection of human small intestinal enterocytes. Sci Immunol 5:eabc3582. doi:10.1126/sciimmunol.abc3582. 32404436 PMC7285829

[13] Radzikowska U, Ding M, Tan G, Zhakparov D, Peng Y, Wawrzyniak P, Wang M, Li S, Morita H, Altunbulakli C, Reiger M, Neumann AU, Lunjani N, Traidl-Hoffmann C, Nadeau K, O’Mahony L, Akdis CA, Sokolowska M. 2020. Distribution of ACE2, CD147, CD26 and other SARS-CoV-2 associated molecules in tissues and immune cells in health and in asthma, COPD, obesity, hypertension, and COVID-19 risk factors. Allergy 75:2829–2845. doi:10.1111/all.14429. 32496587 PMC7300910

[14] Ulrich H, Pillat MM. 2020. CD147 as a target for COVID-19 treatment: suggested effects of azithromycin and stem cell engagement. Stem Cell Rev Rep 16:434–440. doi:10.1007/s12015-020-09976-7. 32307653 PMC7167302

[15] Zhou H, Fang Y, Xu T, Ni W-J, Shen A-Z, Meng X-M. 2020. Potential therapeutic targets and promising drugs for combating SARS-CoV-2. Br J Pharmacol 177:3147–3161. doi:10.1111/bph.15092. 32368792 PMC7267399

[16] Ilikci Sagkan R, Akin-Bali DF. 2020. Structural variations and expression profiles of the SARS-CoV-2 host invasion genes in lung cancer. J Med Virol doi:10.1002/jmv.26107. PMC7300553 32492203

[17] Ahmetaj-Shala B, Vaja R, Atanur SS, George PM, Kirkby NS, Mitchell JA. 2020. Cardiorenal tissues express SARS-CoV-2 entry genes and basigin (BSG/CD147) increases with age in endothelial cells. JACC Basic Transl Sci doi:10.1016/j.jacbts.2020.09.010. PMC7546186 33073064

[18] Matusiak M, Schürch CM. 2020. Expression of SARS-CoV-2 entry receptors in the respiratory tract of healthy individuals, smokers and asthmatics. Respir Res 21:252. doi:10.1186/s12931-020-01521-x. 32993656 PMC7523260

[19] Latini A, Agolini E, Novelli A, Borgiani P, Giannini R, Gravina P, Smarrazzo A, Dauri M, Andreoni M, Rogliani P, Bernardini S, Helmer-Citterich M, Biancolella M, Novelli G. 2020. COVID-19 and genetic variants of protein involved in the SARS-CoV-2 entry into the host cells. Genes (Basel) 11:1010. doi:10.3390/genes11091010. PMC7565048 32867305

[20] Singh M, Bansal V, Feschotte C. 2020. A single-cell RNA expression map of human coronavirus entry factors. Cell Rep 32:108175. doi:10.1016/j.celrep.2020.108175. 32946807 PMC7470764

[21] Zamorano Cuervo N, Grandvaux N. 2020. ACE2: evidence of role as entry receptor for SARS-CoV-2 and implications in comorbidities. Elife 9:e61390. doi:10.7554/eLife.61390. 33164751 PMC7652413

[22] Muramatsu T. 2016. Basigin (CD147), a multifunctional transmembrane glycoprotein with various binding partners. J Biochem 159:481–490. doi:10.1093/jb/mvv127. 26684586 PMC4846773

[23] Wright KE, Hjerrild KA, Bartlett J, Douglas AD, Jin J, Brown RE, Illingworth JJ, Ashfield R, Clemmensen SB, de Jongh WA, Draper SJ, Higgins MK. 2014. Structure of malaria invasion protein RH5 with erythrocyte basigin and blocking antibodies. Nature 515:427–430. doi:10.1038/nature13715. 25132548 PMC4240730

[24] Crosnier C, Bustamante LY, Bartholdson SJ, Bei AK, Theron M, Uchikawa M, Mboup S, Ndir O, Kwiatkowski DP, Duraisingh MT, Rayner JC, Wright GJ. 2011. Basigin is a receptor essential for erythrocyte invasion by Plasmodium falciparum. Nature 480:534–537. doi:10.1038/nature10606. 22080952 PMC3245779

[25] Reference deleted.

[26] Yuan M, Wu NC, Zhu X, Lee C-CD, So RTY, Lv H, Mok CKP, Wilson IA. 2020. A highly conserved cryptic epitope in the receptor binding domains of SARS-CoV-2 and SARS-CoV. Science 368:630–633. doi:10.1126/science.abb7269. 32245784 PMC7164391

[27] Wang C, Li W, Drabek D, Okba NMA, van Haperen R, Osterhaus ADME, van Kuppeveld FJM, Haagmans BL, Grosveld F, Bosch B-J. 2020. A human monoclonal antibody blocking SARS-CoV-2 infection. Nat Commun 11:2251. doi:10.1038/s41467-020-16256-y. 32366817 PMC7198537

[28] Alsoussi WB, Turner JS, Case JB, Zhao H, Schmitz AJ, Zhou JQ, Chen RE, Lei T, Rizk AA, McIntire KM, Winkler ES, Fox JM, Kafai NM, Thackray LB, Hassan AO, Amanat F, Krammer F, Watson CT, Kleinstein SH, Fremont DH, Diamond MS, Ellebedy AH. 2020. A potently neutralizing antibody protects mice against SARS-CoV-2 infection. J Immunol 205:915–922. doi:10.4049/jimmunol.2000583. 32591393 PMC7566074

[29] Shilts J, Crozier TWM, Greenwood EJD, Lehner PJ, Wright GJ. 2021. No evidence for basigin/CD147 as a direct SARS-CoV-2 spike binding receptor. Sci Rep 11:413. doi:10.1038/s41598-020-80464-1. 33432067 PMC7801465

[30] Bian H, Zheng Z-H, Wei D, Wen A, Zhang Z, Lian J-Q, Kang W-Z, Hao C-Q, Wang J, Xie R-H, Dong K, Xia J-L, Miao J-L, Kang W, Li G, Zhang D, Zhang M, Sun X-X, Ding L, Zhang K, Jia J, Ding J, Li Z, Jia Y, Liu L-N, Zhang Z, Gao Z-W, Du H, Yao N, Wang Q, Wang K, Geng J-J, Wang B, Guo T, Chen R, Zhu Y-M, Wang L-J, He Q, Yao R-R, Shi Y, Yang X-M, Zhou J-S, Ma Y-N, Wang Y-T, Liang X, Huo F, Wang Z, Zhang Y, Yang X, Zhang Y, et al. 2021. Safety and efficacy of meplazumab in healthy volunteers and COVID-19 patients: a randomized phase 1 and an exploratory phase 2 trial. Signal Transduct Target Ther 6:194. doi:10.1038/s41392-021-00603-6. 34001849 PMC8127508

[31] Liao C-G, Kong L-M, Song F, Xing J-L, Wang L-X, Sun Z-J, Tang H, Yao H, Zhang Y, Wang L, Wang Y, Yang X-M, Li Y, Chen Z-N. 2011. Characterization of basigin isoforms and the inhibitory function of basigin-3 in human hepatocellular carcinoma proliferation and invasion. Mol Cell Biol 31:2591–2604. doi:10.1128/MCB.05160-11. 21536654 PMC3133368

[32] Peng C, Zhang S, Lei L, Zhang X, Jia X, Luo Z, Huang X, Kuang Y, Zeng W, Su J, Chen X. 2017. Epidermal CD147 expression plays a key role in IL-22-induced psoriatic dermatitis. Sci Rep 7:44172. doi:10.1038/srep44172. 28272440 PMC5341158

[33] Wang Q, Xu B, Fan K, Wu J, Wang T. 2020. Inflammation suppression by dexamethasone via inhibition of CD147-mediated NF-κB pathway in collagen-induced arthritis rats. Mol Cell Biochem doi:10.1007/s11010-020-03808-5. 32594339

[34] Jin R, Zhong W, Liu S, Li G. 2019. CD147 as a key mediator of the spleen inflammatory response in mice after focal cerebral ischemia. J Neuroinflammation 16:198. doi:10.1186/s12974-019-1609-y. 31666088 PMC6822438

[35] Supper V, Schiller HB, Paster W, Forster F, Boulègue C, Mitulovic G, Leksa V, Ohradanova-Repic A, Machacek C, Schatzlmaier P, Zlabinger GJ, Stockinger H. 2016. Association of CD147 and calcium exporter PMCA4 uncouples IL-2 expression from early TCR signaling. J Immunol 196:1387–1399. doi:10.4049/jimmunol.1501889. 26729804

[36] Dawar FU, Xiong Y, Khattak MNK, Li J, Lin L, Mei J. 2017. Potential role of cyclophilin A in regulating cytokine secretion. J Leukoc Biol 102:989–992. doi:10.1189/jlb.3RU0317-090RR. 28729360

[37] Douglas AD, Williams AR, Knuepfer E, Illingworth JJ, Furze JM, Crosnier C, Choudhary P, Bustamante LY, Zakutansky SE, Awuah DK, Alanine DG, Theron M, Worth A, Shimkets R, Rayner JC, Holder AA, Wright GJ, Draper SJ. 2014. Neutralization of Plasmodium falciparum merozoites by antibodies against PfRH5. J Immunol 192:245–258. doi:10.4049/jimmunol.1302045. 24293631 PMC3872115

[38] Zenonos ZA, Dummler SK, Müller-Sienerth N, Chen J, Preiser PR, Rayner JC, Wright GJ. 2015. Basigin is a druggable target for host-oriented antimalarial interventions. J Exp Med 212:1145–1151. doi:10.1084/jem.20150032. 26195724 PMC4516795

[39] Amanat F, Stadlbauer D, Strohmeier S, Nguyen THO, Chromikova V, McMahon M, Jiang K, Arunkumar GA, Jurczyszak D, Polanco J, Bermudez-Gonzalez M, Kleiner G, Aydillo T, Miorin L, Fierer DS, Lugo LA, Kojic EM, Stoever J, Liu STH, Cunningham-Rundles C, Felgner PL, Moran T, García-Sastre A, Caplivski D, Cheng AC, Kedzierska K, Vapalahti O, Hepojoki JM, Simon V, Krammer F. 2020. A serological assay to detect SARS-CoV-2 seroconversion in humans. Nat Med 26:1033–1036. doi:10.1038/s41591-020-0913-5. 32398876 PMC8183627

[40] Jin J, Hjerrild KA, Silk SE, Brown RE, Labbe GM, Marshall JM, Wright KE, Bezemer S, Clemmensen SB, Biswas S, Li Y, El-Turabi A, Douglas AD, Hermans P, Detmers FJ, de Jongh WA, Higgins MK, Ashfield R, Draper SJ. 2017. Accelerating the clinical development of protein-based vaccines for malaria by efficient purification using a four amino acid C-terminal “C-tag.” Int J Parasitol 47:435–446. doi:10.1016/j.ijpara.2016.12.001. 28153778 PMC5482323

[41] Alanine DGW, Quinkert D, Kumarasingha R, Mehmood S, Donnellan FR, Minkah NK, Dadonaite B, Diouf A, Galaway F, Silk SE, Jamwal A, Marshall JM, Miura K, Foquet L, Elias SC, Labbé GM, Douglas AD, Jin J, Payne RO, Illingworth JJ, Pattinson DJ, Pulido D, Williams BG, de Jongh WA, Wright GJ, Kappe SHI, Robinson CV, Long CA, Crabb BS, Gilson PR, Higgins MK, Draper SJ. 2019. Human antibodies that slow erythrocyte invasion potentiate malaria-Neutralizing Antibodies. Cell 178:216–228.e21. doi:10.1016/j.cell.2019.05.025. 31204103 PMC6602525

[42] Hjerrild KA, Jin J, Wright KE, Brown RE, Marshall JM, Labbé GM, Silk SE, Cherry CJ, Clemmensen SB, Jørgensen T, Illingworth JJ, Alanine DGW, Milne KH, Ashfield R, de Jongh WA, Douglas AD, Higgins MK, Draper SJ. 2016. Production of full-length soluble Plasmodium falciparum RH5 protein vaccine using a Drosophila melanogaster Schneider 2 stable cell line system. Sci Rep 6:30357. doi:10.1038/srep30357. 27457156 PMC4960544

[43] Malkin EM, Diemert DJ, McArthur JH, Perreault JR, Miles AP, Giersing BK, Mullen GE, Orcutt A, Muratova O, Awkal M, Zhou H, Wang J, Stowers A, Long CA, Mahanty S, Miller LH, Saul A, Durbin AP. 2005. Phase 1 clinical trial of apical membrane antigen 1: an asexual blood-stage vaccine for Plasmodium falciparum malaria. Infect Immun 73:3677–3685. doi:10.1128/IAI.73.6.3677-3685.2005. 15908397 PMC1111886

