# Supplementary figures and images for: Human Basigin (CD147) Does Not Directly Interact with SARS-CoV-2 Spike Glycoprotein
Source: mSphere. 2021 Aug 11;6(4):e00647-21. doi: 10.1128/mSphere.00647-21 (PMC8386461; doi:10.1128/mSphere.00647-21)

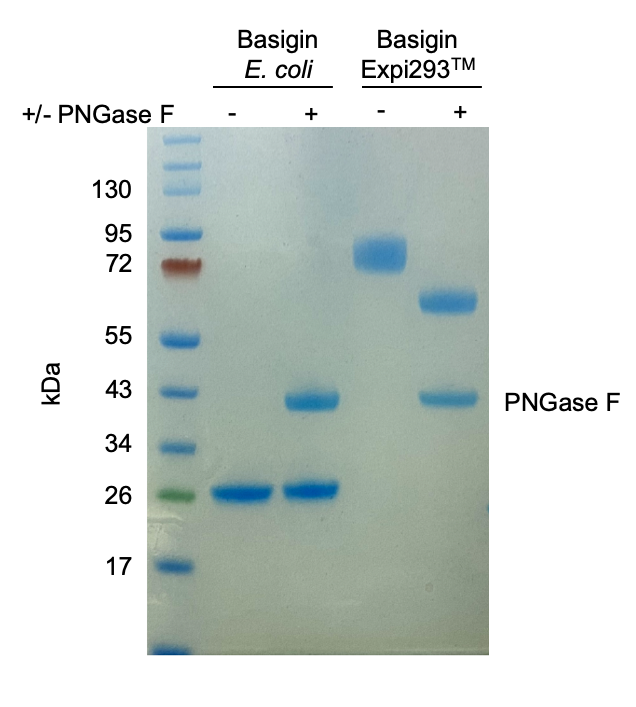

Supplement: FIG S1 [file msphere.00647-21-sf001.tif]

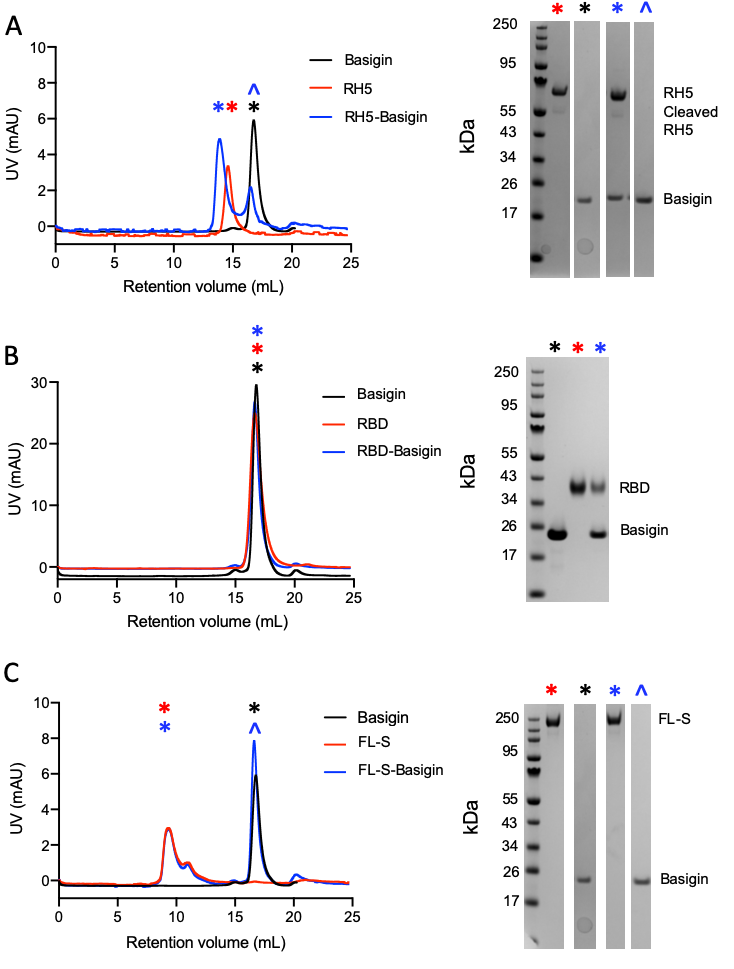

Supplement: FIG S2 [file msphere.00647-21-sf002.tif]
